# Supplementary material for: Fluid overload is a determinant for cardiac structural and functional impairments in type 2 diabetes mellitus and chronic kidney disease stage 5 not undergoing dialysis
Source: PLoS One. 2020 Jul 30;15(7):e0235640. doi: 10.1371/journal.pone.0235640 (PMC7392282; doi:10.1371/journal.pone.0235640)
Supplement: S1 Table — (DOCX) [file pone.0235640.s001.docx]

S1 Table. Comparison of demographics, serum chemistry, echocardiographic findings, and volume status between patients with and without left ventricular diastolic dysfunction.

| Variables | No LVDD (n=65) | LVDD (n=70) | *P*-value |
| --- | --- | --- | --- |
| Age, years | 59.62±11.15 | 60.67±12.25 | 0.602 |
| SBP, mmHg | 141.02±20.48 | 148.50±16.09 | 0.022 |
| DBP, mmHg | 80.83±10.57 | 79.71±9.88 | 0.532 |
| cBMI, kg/m^2^ | 23.73±4.21 | 24.41±4.19 | 0.355 |
| LAD, cm | 4.46±0.43 | 4.79±0.45 | <0.001 |
| LAVI, mL/m^2^ | 33.86±7.68 | 40.46±10.97 | <0.001 |
| E/e´ ratio | 12.25±2.38 | 19.89±4.76 | <0.001 |
| LVEDD, cm | 5.29±0.59 | 5.43±0.47 | 0.124 |
| LVEDV, mL | 138.95±32.75 | 143.30±33.40 | 0.448 |
| LVMI, g/m^2^ | 108.14±24.25 | 119.11±21.46 | 0.006 |
| RWT | 0.36±0.06 | 0.36±0.06 | 0.945 |
| LVEF, % | 63.48±5.46 | 63.17±5.15 | 0.739 |
| NT-proBNP, pg/mL* | 1,815  (455 ~ 6,932) | 3,813  (1,055 ~ 11,018) | 0.003 |
| hs-CRP, mg/dL | 1.32±2.81 | 1.37±3.19 | 0.921 |
| iPTH, pg/mL | 254.06±128.02 | 292.20±177.62 | 0.160 |
| HbA1C, % | 6.93±1.69 | 7.01±1.67 | 0.810 |
| Hemoglobin, g/dL | 9.25±1.34 | 8.99±1.18 | 0.221 |
| Total protein, g/dL | 6.11±0.81 | 6.08±0.81 | 0.836 |
| Albumin, g/dL | 3.43±0.58 | 3.38±0.57 | 0.598 |
| Total cholesterol, mg/dL | 145.73±41.09 | 148.53±42.15 | 0.699 |
| HDL-C, mg/dL | 39.19±13.21 | 37.94±12.63 | 0.581 |
| LDL-C, mg/dL | 80.38±35.94 | 84.00±36.60 | 0.569 |
| Triglyceride, mg/dL | 135.95±62.09 | 132.06±48.86 | 0.686 |
| Calcium, mg/dL | 7.83±0.97 | 7.76±0.93 | 0.665 |
| Phosphate, mg/dL | 5.80±1.64 | 5.97±1.26 | 0.499 |
| eGFR, mL/min/1.73 m^2^ | 7.55±2.53 | 6.70±2.18 | 0.038 |
| OH, liter | 2.98±2.83 | 4.08±3.53 | 0.053 |
| OH/ECW, % | 15.50±12.64 | 20.94±14.57 | 0.025 |
| ECW/TBW | 0.50±0.04 | 0.52±0.04 | 0.001 |

* Mann-Whitney *U* test; median (interquartile range)

cBMI, corrected body mass index; DBP, diastolic blood pressure; ECW, extracellular water; eGFR, estimated glomerular filtration rate; HDL-C, high-density lipoprotein cholesterol; hs-CRP, high-sensitivity C-reactive protein; iPTH, intact parathyroid hormone; LAD, left atrial dimension; LAVI, left atrial volume index; LDL-C, low-density lipoprotein cholesterol; LVEDD, left ventricular end-diastolic dimension; LVEF, left ventricular ejection fraction; LVEDV, left ventricular end-diastolic volume; LVH, left ventricular hypertrophy; LVMI, left ventricular mass index; NT-proBNP, N-terminal prohormone of B-type natriuretic peptide; OH, overhydration; RWT, relative wall thickness; SBP, systolic blood pressure; TBW, total body water.
