# Supplementary material for: Fluid overload is a determinant for cardiac structural and functional impairments in type 2 diabetes mellitus and chronic kidney disease stage 5 not undergoing dialysis
Source: PLoS One. 2020 Jul 30;15(7):e0235640. doi: 10.1371/journal.pone.0235640 (PMC7392282; doi:10.1371/journal.pone.0235640)
Supplement: S2 Table — (DOCX) [file pone.0235640.s002.docx]

S2 Table. Comparison of demographics, serum chemistry, echocardiographic findings, and volume status between patients with and without left ventricular hypertrophy.

| Variables | No LVH(n=56) | LVH (n=79) | *P*-value |
| --- | --- | --- | --- |
| Age, years | 59.96±11.29 | 60.30±12.06 | 0.869 |
| SBP, mmHg | 142.11±20.69 | 146.92±16.86 | 0.146 |
| DBP, mmHg | 81.73±11.19 | 79.17±9.35 | 0.158 |
| cBMI, kg/m^2^ | 24.13±4.16 | 24.04±4.25 | 0.908 |
| LAD, cm | 4.46±0.47 | 4.75±0.44 | <0.001 |
| LAVI, mL/m^2^ | 31.98±7.31 | 41.04±10.08 | <0.001 |
| E/e´ ratio | 13.75±3.53 | 17.96±5.80 | <0.001 |
| LVEDD, cm | 5.18±0.53 | 5.50±0.50 | 0.001 |
| LVEDV, mL | 131.89±27.34 | 147.81±35.24 | 0.005 |
| LVMI, g/m^2^ | 94.27±13.61 | 127.70±18.49 | <0.001 |
| RWT | 0.34±0.06 | 0.37±0.06 | 0.008 |
| LVEF, % | 62.96±5.01 | 63.57±5.49 | 0.514 |
| NT-proBNP, pg/mL* | 793  (391 ~ 3,427) | 4,398  (1,959 ~ 10,577) | <0.001 |
| hs-CRP, mg/dL | 1.20±2.39 | 1.44±3.40 | 0.662 |
| iPTH, pg/mL | 260.97±156.15 | 283.33±157.11 | 0.417 |
| HbA1C, % | 7.22±2.08 | 6.80±1.29 | 0.210 |
| Hemoglobin, g/dL | 9.39±1.30 | 8.92±1.20 | 0.032 |
| Total protein, g/dL | 6.39±0.86 | 5.88±0.70 | <0.001 |
| Albumin, g/dL | 3.63±0.56 | 3.24±0.52 | <0.001 |
| Total cholesterol, mg/dL | 142.30±39.30 | 150.71±42.94 | 0.249 |
| HDL-C, mg/dL | 38.22±13.99 | 38.78±12.10 | 0.808 |
| LDL-C, mg/dL | 76.22±36.41 | 86.63±37.70 | 0.104 |
| Triglyceride, mg/dL | 141.29±65.71 | 128.63±46.35 | 0.193 |
| Calcium, mg/dL | 8.11±0.91 | 7.58±0.91 | 0.001 |
| Phosphate, mg/dL | 5.49±1.44 | 6.18±1.40 | 0.006 |
| eGFR, mL/min/1.73 m^2^ | 7.84±2.52 | 6.60±2.16 | 0.003 |
| OH, liter | 2.34±2.71 | 4.43±3.34 | <0.001 |
| OH/ECW, % | 12.44±13.00 | 22.58±13.00 | <0.001 |
| ECW/TBW | 0.49±0.04 | 0.52±0.04 | <0.001 |

* Mann-Whitney *U* test; median (interquartile range)

cBMI, corrected body mass index; DBP, diastolic blood pressure; ECW, extracellular water; eGFR, estimated glomerular filtration rate; HDL-C, high-density lipoprotein cholesterol; hs-CRP, high-sensitivity C-reactive protein; iPTH, intact parathyroid hormone; LAD, left atrial dimension; LAVI, left atrial volume index; LDL-C, low-density lipoprotein cholesterol; LVEDD, left ventricular end-diastolic dimension; LVEF, left ventricular ejection fraction; LVEDV, left ventricular end-diastolic volume; LVH, left ventricular hypertrophy; LVMI, left ventricular mass index; NT-proBNP, N-terminal prohormone of B-type natriuretic peptide; OH, overhydration; RWT, relative wall thickness; SBP, systolic blood pressure; TBW, total body water.
