# Supplementary material for: Fluid overload is a determinant for cardiac structural and functional impairments in type 2 diabetes mellitus and chronic kidney disease stage 5 not undergoing dialysis
Source: PLoS One. 2020 Jul 30;15(7):e0235640. doi: 10.1371/journal.pone.0235640 (PMC7392282; doi:10.1371/journal.pone.0235640)
Supplement: S3 Table — (DOCX) [file pone.0235640.s003.docx]

S3 Table**.** Stepwise multiple linear regression of variables associated with E/e′ ratio and left ventricular mass index.

|  | E/e′ ratio * | | LVMI * | |
| --- | --- | --- | --- | --- |
|  | Β (95% CI) | *P*-value | Β (95% CI) | *P*-value |
| cBMI, kg/m^2^ | 0.309 (0.100, 0.518) | 0.004 | 1.080 (0.100, 2.061) | 0.031 |
| OH/ECW, % | 0.141 (0.076, 0.205) | <0.001 | 0.519 (0.202, 0.836) | 0.002 |
| Female (vs. male) | 3.545 (1.792, 5.298) | <0.001 |  |  |
| SBP, mmHg | 0.068 (0.022, 0.114) | 0.004 |  |  |
| Calcium, mg/dL |  |  | -5.755 (-10.163, -1.347) | 0.011 |

Only significant determinants were presented.

*Adjusted for: age, hs-CRP, albumin, phosphorus, and eGFR.

B, β coefficient; cBMI, corrected body mass index; CI, confidence interval; ECW, extracellular water; eGFR, estimated glomerular filtration rate; hs-CRP, high-sensitivity C reactive protein; LVMI, left ventricular mass index; OH, overhydration; SBP, systolic blood pressure.
