# Supplementary material for: Fluid overload is a determinant for cardiac structural and functional impairments in type 2 diabetes mellitus and chronic kidney disease stage 5 not undergoing dialysis
Source: PLoS One. 2020 Jul 30;15(7):e0235640. doi: 10.1371/journal.pone.0235640 (PMC7392282; doi:10.1371/journal.pone.0235640)
Supplement: Attachment — Submitted filename: Response to Reviewers.docx [file pone.0235640.s004.docx]

*June 06, 2020*

*Dear Prof. Vincenzo Lionetti:*

We would like to thank you and the reviewers of the *PLOS ONE* for taking the time to review our article *(PONE-D-20-08628)*. We have made corrections and clarifications in the manuscript after going over the reviewers’ comments. The changes are summarized below:

***Reviewer #1:*** Dear Authors,

it is an interesting manuscript that offers sound information about the effects of fluids overload on the heart in type2 DM.

it is well written and straightforward.

I have only a couple of observations:

In abstract you write that measurements were performed "on the same day". In methods it is not clear when the measurement were done (prior dyalisis). May you better explain it ?

🡪 Description regarding the time of echocardiographic study was added as you pointed out. Bioimpedance spectroscopy (BIS), echocardiography, serum sampling, and etc. should be performed at the same time.

What the story before the day of measurement ? how long patients have suffered DM, how long the story of CKD ?

🡪 Thank you for your valuable comments. We understand why you left this comment. We had the same above-mentioned concerns during our analysis. Our hospital is a tertiary institution in a small city. Approximately 50 patients were enrolled in our study each year. After our patients were ready for renal replacement therapy such as arteriovenous fistula formation, we transferred them back to their hometowns. In general, most of the patients’ residential areas are rural areas that are smaller than the city of our institution. Therefore, it was often meaningless to use patients’ own account of disease duration for analysis because they did not know exactly when their diabetes or chronic kidney disease begun. In addition, it is difficult to check up on the patient's outcomes after getting transferred. This can be said to be one of the limitations of our research.

Another aspect needs some information. Volume status was calculated with bioimpedenzometry that is affected by BMI. You somehow corrected this, but it could be interesting to have the BMI of the evaluated patients and a discussion on the possible bias caused by BMI in the analysis. There were differences between patients with low and high BMI ?

🡪 As you know, there is no way to accurately evaluate the volume status that is not affected by BMI. We are well aware that there are many limitations of bioimpedance measurement. However, we think that bioimpedance measurement may be one of the useful tools in clinical practice. Therefore, evaluation of factors affecting between volume status and BMI is considered to be beyond the scope of this study and may be the subject of future research. For reference, our patients tended to increase in volume status as BMI increased.

In the equation “cBMI=(weight-OH)/height^2^”, the part “(weight-OH)” describes the normohydrated weight that the patient would have with a working kidney. We thought that it could provide a possibility to make it comparable with healthy subjects. We have not found any form of research attempted with this calibration method in previous studies.

It is true that the three compartments of BIS model contributed to the evaluation and diagnosis of the nutritional and hydration status of patients with end-stage renal disease by subdividing the body compartment. On the other hand, because BMI does not subdivide body compartments and evaluate them, there are limitations in evaluating patients with fluid overload. Traditionally, BMI is focused on nutritional status, and its role in patients with fluid overload is relatively less defined. In the epidemiologic study of the general population, BMI is well known as a tool for predicting prognosis, but it does not accurately reflect fluid imbalance in patients with end-stage renal failure requiring dialysis. Since BMI does not accurately reflect the nutritional and/or volume status in patients with ESRD, it is necessary to evaluate the body composition separately. The cBMI mentioned in our study was simply the first attempt to address this inaccuracy. In another study that we are currently working on, we have confirmed that there is a difference between BMI and cBMI and need further analysis. We want to show our ROC data for reference.

|  | AUC | SE | *P*-value | 95% CI |
| --- | --- | --- | --- | --- |
| BMI, kg/m^2^ | 0.638 | 0.051 | 0.010 | 0.538, 0.738 |
| cBMI, kg/m^2^ | 0.689 | 0.049 | <0.001 | 0.594, 0.784 |

Your valuable comments make us feel that further analysis regarding volume status and BMI is needed in the future as a new project. We would like to say thank you again for your point.

minor: in § "assessment of the volume status" explanation of BIS and BCM are lacking.

🡪 We have further described what you pointed out.

***Reviewer #2:*** In this paper, the authors want to investigate the association between fluid overload and left ventricular hypertrophy and left ventricular diastolic dysfunction in patient with DM type 2 and stage 5 chronic kidney disease. The manuscript is fairly well written, flow and readability are good. The rationale of the paper is clear and the analysis is sound. I consider the manuscript a good piece of research that improves the understanding of an interesting subject.
